# Riluzole: a potential therapeutic intervention in human brain tumor stem-like cells

**DOI:** 10.18632/oncotarget.18043

**Published:** 2017-05-20

**Authors:** Swetlana Sperling, Thiha Aung, Sabine Martin, Veit Rohde, Milena Ninkovic

**Affiliations:** ^1^ The Translational Neurooncology Research Group, Department of Neurosurgery, University Medical Center Göttingen, University Göttingen, Göttingen, Germany; ^2^ Center of Plastic, Hand and Reconstructive Surgery, University Medical Center Regensburg, Regensburg, Germany; ^3^ Department of Molecular Biology of Neuronal Signals, Max Planck Institute of Experimental Medicine, Göttingen, Germany; ^4^ Center Nanoscale Microscopy and Molecular Physiology of the Brain (CNMPB), Göttingen, Germany

**Keywords:** riluzole, human brain tumor stem-like cells, GLUT3, DNMT1, cell death

## Abstract

A small subpopulation of tumor stem-like cells has the capacity to initiate tumors and mediate radio- and chemoresistance in diverse cancers hence also in glioblastoma (GBM). It has been reported that this capacity of tumor initiation in the brain is mainly dependent on the body’s nutrient supply. This population of so-called brain tumor initiating or brain tumor stem-like cells (BTSCs) is able to extract nutrients like glucose with a higher affinity. Riluzole, a drug approved for treating amyotrophic lateral sclerosis (ALS), was reported to possess anticancer properties, affecting the glutamate metabolism. We report that riluzole treatment inhibits the growth of brain tumor stem-like cells enriched cultures isolated from two human glioblastomas. The effects of riluzole on these cells were associated with an inhibition of a poor prognostic indicator: glucose transporter 3 (GLUT3). A decrease in GLUT3 is associated with a decrease in the p-Akt/HIF1α pathway. Further, downregulation of the DNA (Cytosine-5-)-methyltransferase 1 (DNMT1) gene that causes hypermethylation of various tumor-suppressor genes and leads to a poor prognosis in GBM, was detected. Two hallmarks of cancer cells—proliferation and cell death—were positively influenced by riluzole treatment. Finally, we observed that riluzole reduced the tumor growth in *in vivo* CAM assay, suggesting it could be a possible synergistic drug for the treatment of glioblastoma.

## INTRODUCTION

Metabolic alterations in cancers, which are characterized by cells favoring aerobic glycolysis rather than oxidative phosphorylation, are known as possessing what is called the Warburg effect [1]. Cancer cells are highly dependent on glucose as a carbon source. In glioblastoma (GBM), the most lethal and prevalent primary form of malignant brain tumor, glycolysis was upregulated by as much as threefold compared to a measurement taken from normal brain tissue [2]. As the primary energy source in a normal brain, glucose [3] must be transported across the endothelial cells of the blood-brain barrier and then taken up into the neuronal and glial cells. Two isoforms transport glucose into cells; the glucose transporter proteins 1 and 3 (GLUT1 and GLUT3) [4, 5]. The GLUT1 (gene: SLC2A1) and GLUT3 (gene: SLC2A3) proteins are significant indicators of a poor prognosis outcome in oral squamous cell carcinoma, probably because of the enhanced glycolytic metabolism of more aggressive neoplastic cells [6]. The GLUT3 level in gliomas increases with brain tumor grade, with GBM showing the highest expression, and the expression of GLUT3 is found to be higher in recurrent tumors than in primary tumors [7]. Moreover, it was found that the expression of GLUT3 was also correlated with poor survival with breast, colon, ovary, and lung tumors. GLUT3 expression also contributes to the proliferation of lung tumor cells and is an independent prognostic factor of poor overall survival in non-small cell lung cancers [8].

Stem-like tumor cells are believed to be responsible for tumor initiation in a variety of cancers, hence also in GBM. Brain tumor stem-like cells are very resistant to chemo- and radiotherapy; therefore, they are thought to be responsible for the recurrence of gliomas [9]. The comparison of spheroids formed by rat glioma stem cells and neural stem cells reveal differences in glucose metabolism [10]. Similarly, Zhou et al. observed preferences for hypoxia and a high dependency on glycolysis in U87-derived cancer stem cells [11]. Flavahan et al. [7] used xenografted mice to show that glucose restriction induced an increase in the frequency of brain tumor stem-like cells and tumorsphere-forming cells that are associated with faster growth and reduced survival. They also showed that these cells preferentially express GLUT3 and that targeting GLUT3 inhibits brain tumor stem-like cells. Furthermore, nanoparticles carrying specific siRNA targeting GLUT3 inhibited cell metabolism and the proliferation of glioma stem and bulk cells and inhibited tumor growth in the U87MG xenograft model [12]. Altogether, these findings emphasize that developing GLUT3 inhibitory drugs can be a targeted therapy for the treatment of BTSCs in GBM patients.

Riluzole is an approved drug for amyotrophic lateral sclerosis (ALS) [13] and is under testing in clinical trials as a substance for melanoma therapy. Even though the wide range of riluzole actions has been shown in previous studies, how riluzole functions is not completely understood. There are reports about its anti-glutamatergic pharmacological properties that include the inhibition of glutamate release through inactivation of voltage-dependent ion channels [14, 15], effects on the expression and signal transduction through glutamate receptors [14] and facilitation of astrocytic glutamate uptake [16]. Using a model of the U87 glioblastoma cell line, Yelskaya et al. [17] showed that blocking glutamate release by riluzole inhibits cell proliferation. Additionally, clinical studies suggest riluzole can be used as an antidepressant and anxiolytic-like substance for treatments of depression and anxiety disorders [18]. On the other hand, Chowdhury et al. [19] showed that chronic riluzole treatment enhanced glucose oxidative metabolism and glutamate and glutamine cycling.

In the present study, we examined the potential effect of riluzole on brain tumor stem-like cells. Here, we provide evidence of an additional effect of riluzole on GLUT3 transporter in glioblastoma stem-like cells. Furthermore, riluzole is effective in killing brain tumor stem-like cells *in vitro* and inhibits tumor growth *in vivo.*

## RESULTS

### Riluzole effect on cell death in brain tumor stem-like cells

**Figure 1:**
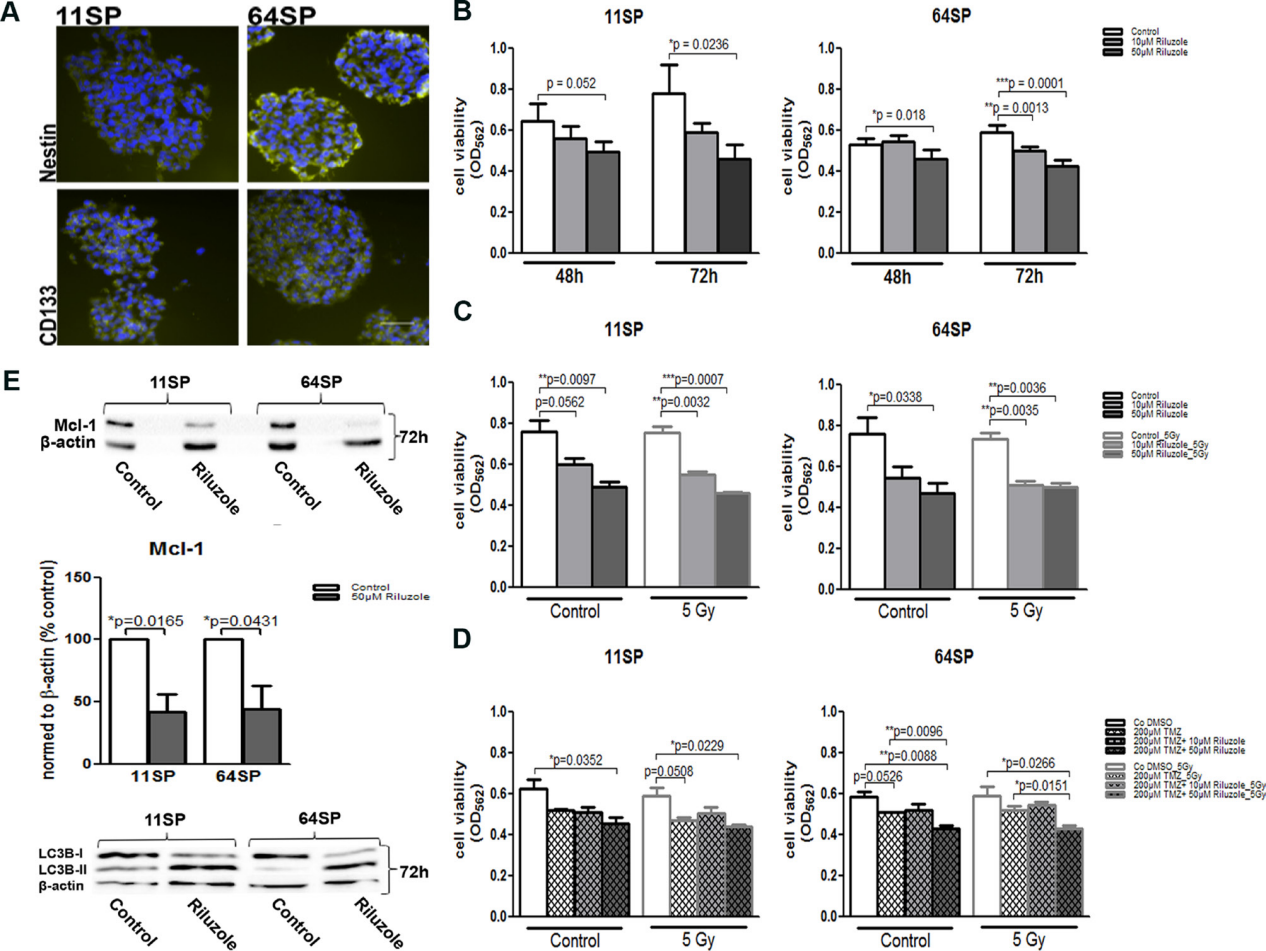
Stem-like properties of BTSCs and its cell viability assessment after the treatment with riluzole (**A**) BTSCs stained with anti-CD133 und anti-Nestin antibodies, known neural stem and progenitor cell markers, in green and DAPI in blue. (**B**) Cell viability obtained by MTT assay (*n* = 5; after 48 and 72 h) after the treatment with 10 µM and 50 µM riluzole alone or in combination with (**C**) irradiation of 5 Gy (*n* = 3; after 72 h) or (**D**) in combination with 200 µM TMZ and irradiation of 5 Gy (*n* = 3; after 72 h). (**E**) A decrease in Mcl-1 protein expression as a consequence of riluzole action was presented by representative western blot with anti-Mcl-1 antibody 72 h after the treatment as well as by densitometry analysis of three independent experiments. Western blot with anti-LC3B antibody shows an increase in LC3B-II and indicates autophagy as a form of cell death. A statistical analysis was performed using two-sided *t*-tests of three independent experiments (^*^p < 0.05, ^**^p < 0.01, ^***^p < 0.001). The scale bar is 50 µm.

In the U87MG glioblastoma cell line riluzole, an approved drug inhibits glutamate-dependent growth [17, 20]. We examined the effect of riluzole on the viability of two brain tumor stem-like cell lines: 11SP and 64SP. Prior to the analysis, we evaluated the BTSCs immunophenotype with CD133, the cell surface marker and Nestin, an intermediate filament protein, known as neural stem and progenitor cell markers (Figure 1A); we also looked at their ability to differentiate into the three different neural lineages and to form colonies in soft agar assay (Supplementary Figure 1) and in *in vivo* CAM assay (Figure 5). MTT assay was performed using two different concentrations—10 µM and 50 µM of riluzole—and was analyzed in a time frame between 48–72 h. The half-maximal concentration (IC50; 50% of growth inhibition) of riluzole on cell lines 11SP and 64SP were determined as > 100 μM (data not shown). The two doses of riluzole, 10 and 50 µM, were chosen because they are within the scale of the maximum tolerated dose of 100 μM in medical practice [21]. The decrease in cell viability was observed as early as 48 h in the presence of riluzole. However, a significant reduction in cell viability was detected using 50 µM riluzole at 72 h (*p* = 0.0236 and *p* = 0.0001) in both cell lines (Figure 1B). The discrepancy observed with the 10 µM dose was most likely because of the unequal number of performed experiments (Figure 1B, 1C). To corroborate our data on radio- and chemosensitivity, we examined the cell viability of cells treated with riluzole and radiotherapy, as well as irradiated cells treated with a combination of riluzole and chemotherapeutic temodal, all at 72 h. Irradiation (5 Gy) in combination with 50 µM riluzole did not show any additional effect, whereas the radiation enhanced the effect of the lower dose of 10 µM riluzole on 11SP cells only (Figure 1C). However, the effect of riluzole together with both temodal and radiotherapy did not show any additional effects (Figure 1D).

One of the connections between metabolism and cell death is the effect of glucose metabolism on the apoptosis. An increase in caspase activity, which would suggest that the cells are dying from apoptosis, could not be detected. Caspase 3 and Caspase 9 expression levels were without change in riluzole-treated cells compared to untreated cells (Supplementary Figure 2). Nonetheless, the level of another key programed cell death protein—myeloid cell leukemia sequence 1 protein (Mcl-1)—was reduced almost 50% after 72 h (Figure 1E). Because Mcl-1 has a second apoptosis-independent role that involves autophagy, we analyzed and detected increased lipidation in the endogenous LC3B protein (Figure 1E), which is an autophagy marker.

### Decrease of proliferation in brain tumor stem-like cells after riluzole treatment

**Figure 2:**
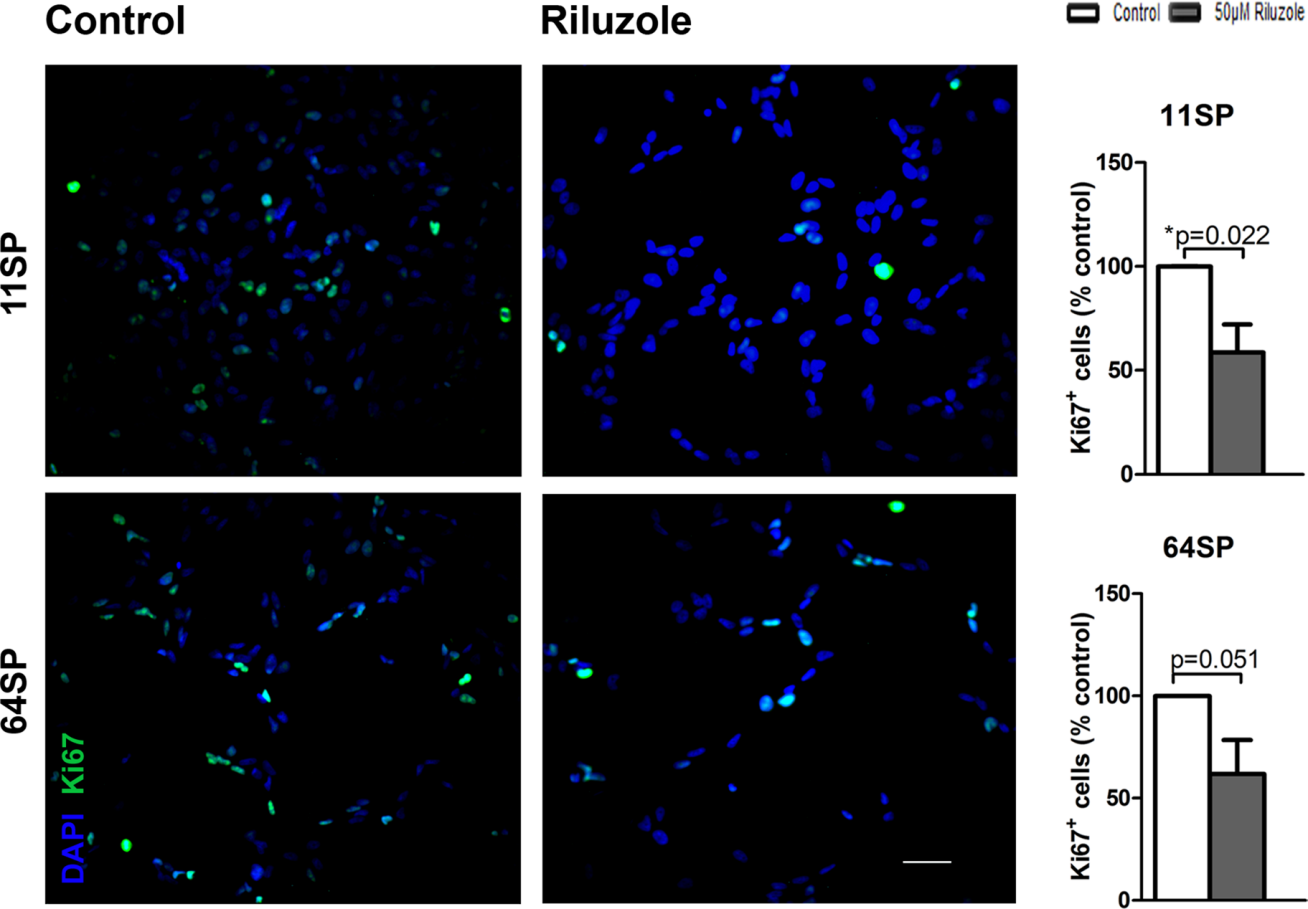
A proliferation analysis of BTSCs after the treatment with riluzole The proliferation of glioblastoma stem-like cells decreases under riluzole treatment in both cell lines. At 24 h after the treatment with 50 µM riluzole, the cells were fixed and stained with anti-Ki-67 antibody. The proportion of Ki-67 positive cells was considerably lower compared to the negative control (*p* = 0.022, *p* = 0.051) in both cell lines. Cell proliferation assays with anti-Ki-67 antibodies were based on at least three independent experiments. Statistical analysis was performed using two-sided *t*-tests for two-group comparisons (^*^p < 0.05, ^**^p < 0.01, ^***^p < 0.001). The scale bar is 50 µm.

Inhibiting the proliferation of glioblastoma cell lines has been reported as an effect of riluzole using a model of U87 glioblastoma cell line and was explained by the known effects of riluzole blocking glutamate release [17]. We tested the influence of riluzole on the proliferation of BTSCs. The percentage of proliferating cells based on the total number of Ki-67 positive cells out of the total number of DAPI positive cells is presented in Figure 2. Compared to the control sample, riluzole-treated samples had a decreased number of both DAPI positive and Ki-67 positive cells (Figure 2). The percentage of proliferating cells declined with riluzole treatment, and riluzole-treated samples were significantly different when compared to the untreated control sample, with *p* = 0.022 and *p* = 0.051. Our results demonstrate that riluzole inhibits the proliferation of two glioblastoma stem-like cell lines.

### Riluzole decreases the expression of glucose transporter 3 in brain tumor stem-like cells

**Figure 3:**
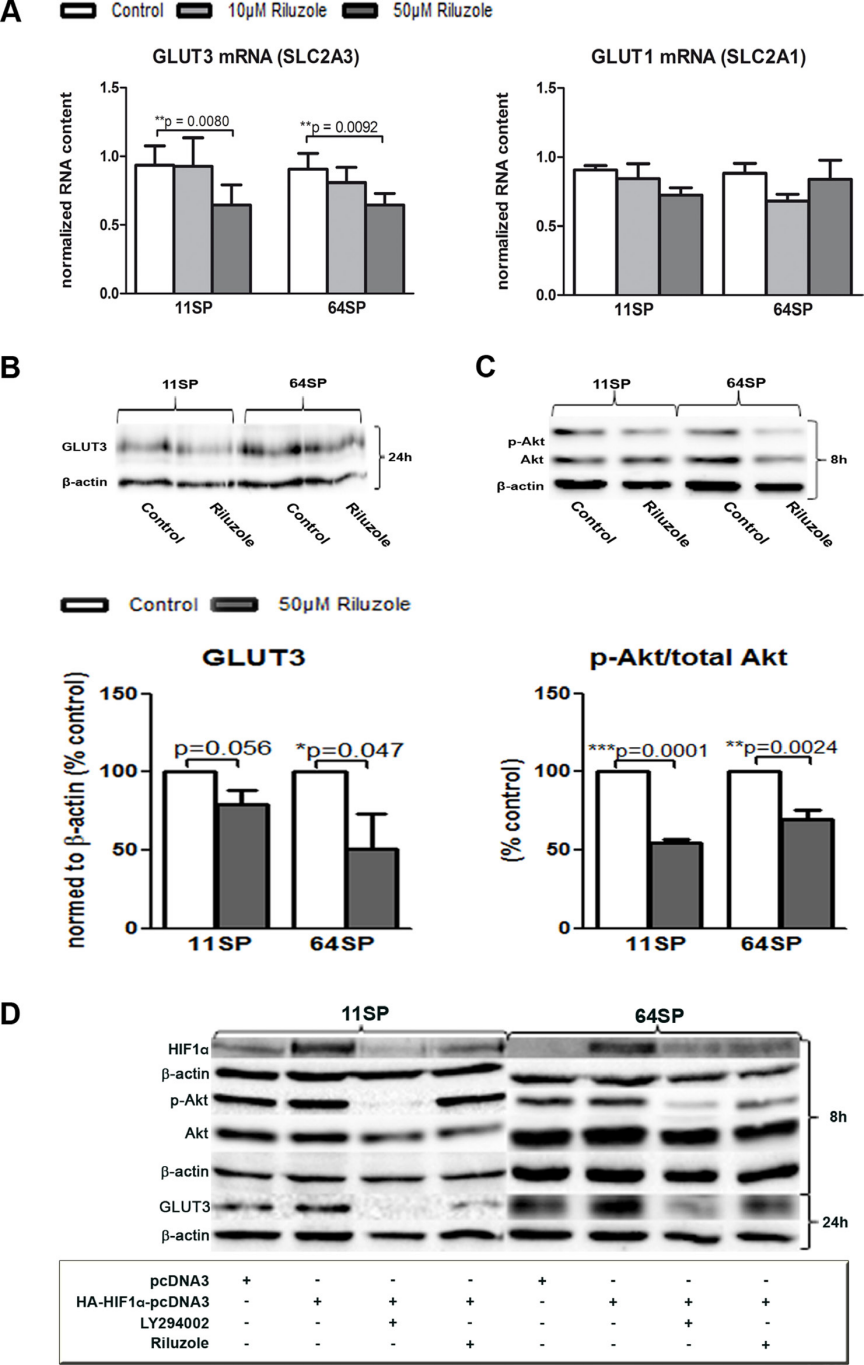
Influence of riluzole on GLUT3 (**A**) A decrease of GLUT3 (SLC2A3), but not GLUT1 (SLC2A1), mRNA expression was seen, as detected by real-time PCR. (**B**) A GLUT3 protein decrease was detected by WB and densitometry (from three independent experiments) analysis after 24 h of treatment with riluzole. (**C**) Western blot analysis of activated Akt (p-Akt) and Akt protein in two BTSC lines after 8 h of treatment with 50 µM riluzole. Representative blots are presented, and statistical analysis of the ratio between Akt and pAkt from three independent experiments is shown. A statistical analysis was performed using two-sided *t*-tests for two-group comparisons (^*^p < 0.05, ^**^p < 0.01, ^***^p < 0.001). (**D**) Inhibitor of PI3K/Akt pathway LY294002, as well as riluzole (50 µM), decreased p-Akt and HIF1α after 8 h and GLUT3 after 24 h. Both cell lines cells were transfected with HA-HIF1α-pcDNA3 [25] or pcDNA3 plasmid alone and treated with either LY294002 or riluzole. Controls of protein loading were immunoblotted with an anti-β-actin antibody.

Here we show that riluzole influences the expression of GLUT3 in two different patient-driven brain tumor stem-like cell lines. Quantitative, real-time PCR was performed 24 h after two BTSCs lines were treated with 10 µM and 50 µM riluzole. 50 µM riluzole decreases mRNA level of SLC2A3 (GLUT3) in both cell lines, where the SLC2A1 mRNA (GLUT1) of another brain glucose transporter was not downregulated (Figure 3A). The inhibition of GLUT3 mRNA, a poor prognostic factor in GBM, is statistically significant; for the cell line 11SP, it was *p* = 0,008 and for 64SP, it was *p* = 0,0092 (Figure 3A). Western blot analysis confirmed that GLUT3 protein expression decreases (Figure 3B) after 24 h treatment with 50 µM of riluzole. This concentration was chosen for all further experiments because it showed a robust effect.

Next, we examined possible molecular mechanisms that cause the reduced GLUT3 expression. Because the Sp1 transcription factor is a positive regulator of GLUT3 expression in human glioma cells [22], at first, we tested its mRNA expression and could not detect any changes when treating with riluzole (data not shown). A lower expression of the glucose transporter leads to a lower glucose uptake, and in the end, this leads to lower metabolism. Glucose is essential for the phosphorylation of Akt, and in turn, Akt has been shown to regulate aspects of cellular metabolism, such as glucose transport [23]. These changes could also be because of the already described mechanism of riluzole, where suppression of the PI3K/Akt/mTOR pathway is a consequence of mGluR1 inhibition. Western blot analysis of physiologically active Akt revealed a reduction of the active form (p-Akt) after exposure of cells to 50 µM riluzole and after only 8 h. The total Akt remained unchanged (Figure 3C). It has been shown that the glucose influence on the phosphorylation of Akt results in an induction of HIF1α expression [23]. However, HIF1α activates the transcription of GLUT3 [24].

To examine if the effect of riluzole on decreased p-Akt expression acts over HIF1α and thereby leads to a reduction of GLUT3 expression, we assessed the effect of PI3K/Akt inhibitor LY294002 on these cells. Additionally, because of the difficulties in detecting the changes in native HIF1α protein concentration, we used an overexpression system. The cells were transfected with either HA-HIF1α-pcDNA3 [25] or pcDNA3 plasmid DNA and treated with LY294002 or riluzole for 8 h (for p-Akt and HIF1α protein analysis), 24 h (for GLUT3), or both. Protein analysis of the group transfected with the HA-HIF1α-pcDNA3 plasmid DNA confirmed HIF1α overexpression (Figure 3D). The samples transfected and treated with either 100 µM LY294002 or 50 µM riluzole showed a decrease in HIF1α. A significant decrease in p-Akt expression was detected in 64SP cells whereas riluzole treated 11SP showed less pronounce effect. GLUT3 protein level decreased in both cell lines and with both treatments (Figure 3D).

### DNMT1 decreases as a consequence of riluzole action

**Figure 4:**
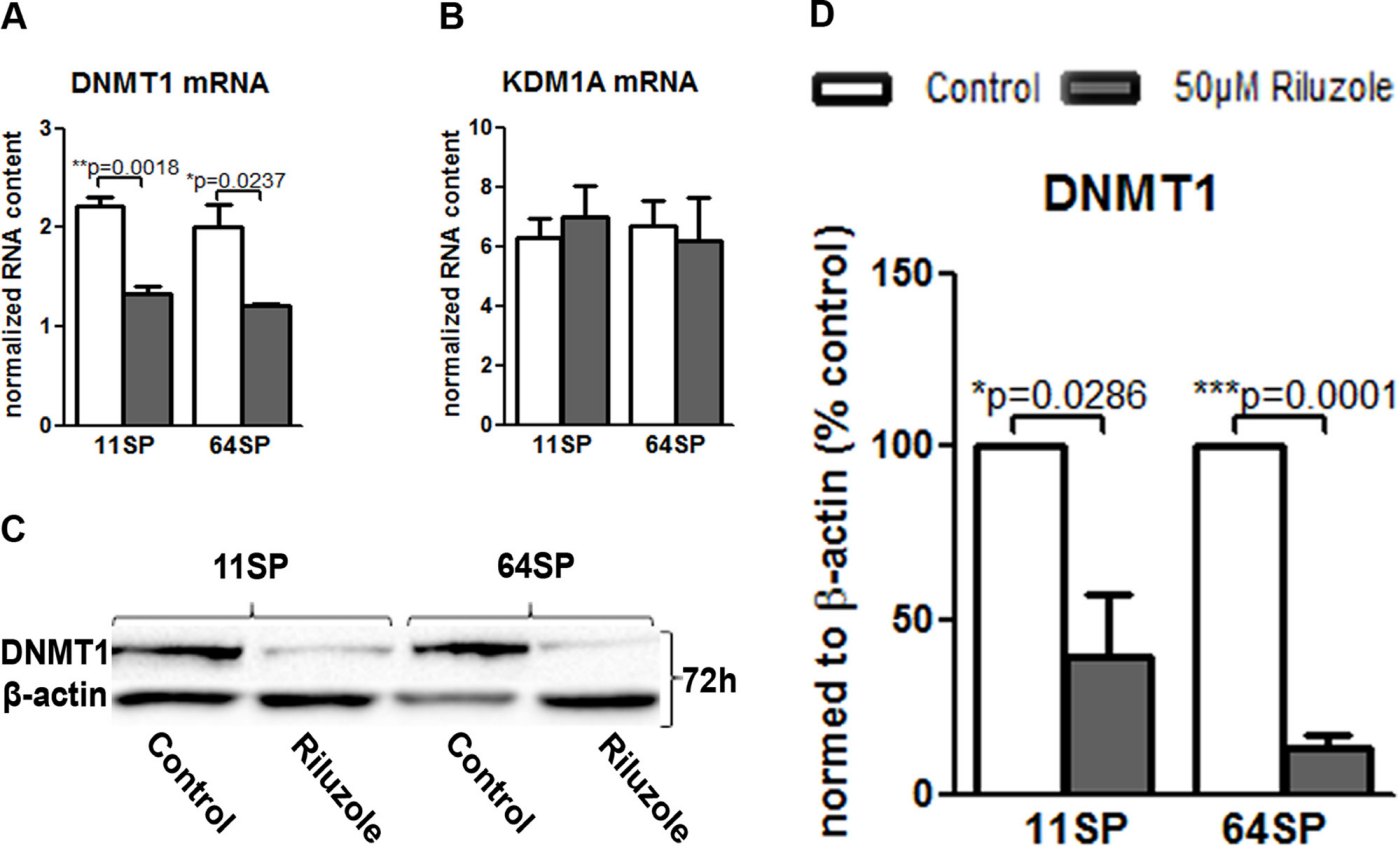
Decrease of DNMT1 after riluzole treatment (**A**) Real-time PCR showed that the treatment of 11SP and 64SP cells with 50 µM riluzole for 72 h decreased DNMT1 mRNA expression. (**B**) KDM1A mRNA level did not change after 72 h. (**C**) A decrease in the DNMT1 protein level was confirmed, which was represented by the western blot with anti-DNMT1 antibody 72 h after the treatment. (**D**) Statistical analysis of DNMT1 protein expression from three independent experiments was performed using two-sided *t*-tests for two-group comparisons (^*^p < 0.05, ^**^p < 0.01, ^***^p < 0.001).

Cell metabolism affects levels of the metabolites that are required substrates of chromatin-modifying enzymes that use these metabolites to post-translationally modify both histones and DNA. The variations in this input determine epigenome remodeling and transcription. In our case, the effect of riluzole on GLUT3 could potentially affect epigenetic changes in these cells. We measured in real-time PCR the effect of riluzole on two enzymes: lysine (K)-specific demethylase 1A (KDM1A) and DNA (cytosine-5-)-methyltransferase 1 (DNMT1). KDM1A was chosen because of its importance for cell survival and self-renewal in sphere formation assays [26]. Furthermore, KDM1A knockdown caused tumor propagating cells to lose their capacity to initiate tumors *in vivo.* DNMT1 is overexpressed in GBM, resulting in a lack of cell growth regulation and higher genomic instability and leading to a poor prognosis in GBM [27]. Figure 4A presents the effect of riluzole on DNMT1 mRNA after 72 h; there was no effect on KDM1A (Figure 4B). Downregulation of DNMT1 has been confirmed in protein levels (Figure 4C and 4D).

### Riluzole treatment of brain tumor stem-like cells reduces tumor formation in the chick chorioallantoic membrane assay (CAM)

Finally, we tested the ability of brain tumor stem-like cell lines to form a solid tumor in the CAM assay. The CAM assay is a well-established model for studying tumors *in vivo* (e.g., as lymphoma [28], prostate cancer [29], ovarian cancer [30], leukemia [31], osteosarcoma [32], and glioma). In this study, 1 × 10^5^ trypsinized cells were implanted on the CAM. The selected GBM BTSC line formed tumors during the first week. The average tumor size was 90.6 mg.

**Figure 5:**
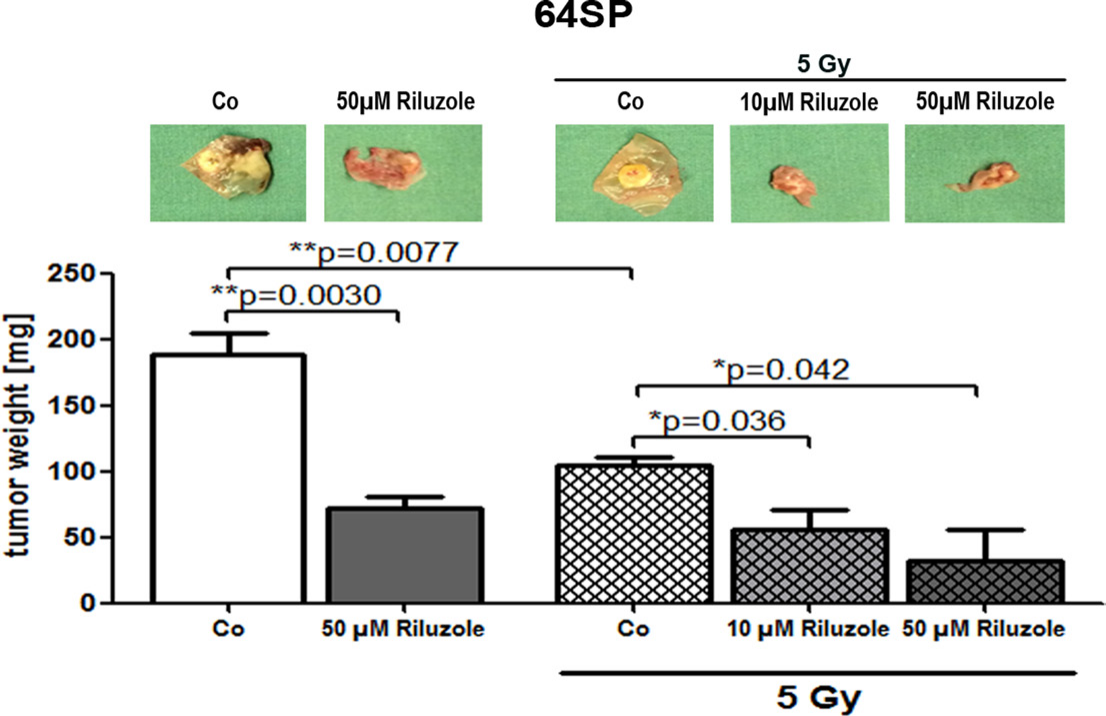
Riluzole reduces tumor formation of GBM stem-like cells in *in vivo* CAM assay Implantation of 64SP trypsinized GBM stem-like cells in CAM assay showed the formation of tumors that had reduced growth after the treatment with 50 µM riluzole. In another set of experiments (3×), the formation of tumors was monitored after the treatment with 10 and 50 µM riluzole in combination with the radiation. The applied dose was 5 Gy. Statistical analysis was performed using two-sided *t*-tests for two-group comparisons (^*^p < 0.05, ^**^p < 0.01, ^***^p < 0.001).

Because all previous experiments showed similar effects on both cell lines, we tested the riluzole effect only on one cell line *in vivo*. One day after implantation, 50 µM riluzole was applied on a marked ring that enclosed the implanted cells. A control treatment of 1X PBS was used. The drug and control treatments were repeated every day. In every experiment, 1-4 CAMs were used for each treatment, and the experiment was repeated three times. Seven days after implantation, the tumors were resected and tumor volumes were measured. Three independent experiments showed that riluzole reduces the formation of tumors *in vivo* (Figure 5). To examine the radiation effect, we repeated the experiment by introducing single radiation of 5 Gy. As seen in Figure 5, the radiation of 5 Gy alone reduces tumor growth, and the combination of radiation and riluzole significantly enhances the growth’s sensitivity to radiation treatment.

## DISCUSSION

Glioblastoma, like many other tumors, displays high rates of glucose uptake, which could indicate that targeting this specific metabolism could be an option for therapy [7]. Flavahan et al. [7] found that brain tumor stem-like cells preferentially express GLUT3 and that targeting GLUT3, but not GLUT1, inhibits BTSCs growth and tumorigenic potential [7, 12].

Riluzole is an approved drug for amyotrophic lateral sclerosis, and its main role is the inhibition of glutamate release, showing riluzole to be a powerful neuroprotective drug. The highly proliferative and aggressive behavior of GBMs has been shown to be due to excessive glutamate release [33], and the inhibition of glioma glutamate release or the blockage of glutamate receptors may serve as an alternative strategy in the management of patients with malignant gliomas [34]. In a U87MG glioblastoma model, blocking glutamate release by riluzole inhibits cell proliferation [17, 20]. This phenomenon has also been described in human melanomas, where riluzole induces apoptosis in mGluR1-expressing melanoma cells [35]. Besides the action of riluzole on mGluR1, riluzole has a wide range of other effects, such as its influence on several ion channels [36, 37] and on heat shock factor 1 [38], which raises the question as to which molecular mechanism riluzole acts through.

The present work was encouraged by the findings of Chowdhury et al. [19], which indicated that contrary to expected action, chronic riluzole enhanced glucose oxidative metabolism and glutamate and glutamine cycling in rat brains. In this study, we showed that riluzole treatment of BTSCs decreases the expression of GLUT3 in two different patient-driven cell lines. A decrease of GLUT3 was shown on mRNA, as well as on the protein level (Figure 3A–3B). This is a very important finding considering that glucose consumption and GLUT3 expression are the drivers of BTSCs and that targeting GLUT3 inhibits BTSCs growth and tumorigenic potential [7, 12]. Increased glucose uptake by the GLUT3 in GBM drives enhanced malignant progression through increased transformation of the tumor cells into stem-like cells. Reducing GLUT3 with riluzole would slow down this effect and also allow for radiotherapy to have a better effect on proliferating cells.

In human glioma cells, the Sp1 transcription factor acts as a positive regulator of GLUT3 expression [22]. This transcription factor is not affected by riluzole treatment. However, the treatment of the BTSCs with riluzole showed a reduction in the levels of biologically active phospho-Akt. A serine/threonine kinase (Akt) supports cell survival and suppresses apoptosis through glucose metabolism [39, 40]. It also promotes the translocation of glucose transporters from the cytosol to the plasma membrane, increasing glucose uptake [40]. The level of activated Akt affects HIF1α protein level [41], and HIF1α activates the transcription of GLUT1 and GLUT3 [24, 42].

To see if the decline of p-Akt after riluzole treatment could cause changes in HIF1α and on GLUT3 was tested in the HIF1α overexpressing system. Cells overexpressing the HIF1α protein were treated by LY294002, a known inhibitor of (PI3K)/Akt/mTOR (reviewed in [43]). Both cell lines showed downregulation of HIF1α and p-Akt, as expected. The similar effect was observed in riluzole treatment of 64SP whereas 11SP did not show obvious downregulation of p-Akt. Because the level of activated Akt has an effect on the HIF1α protein level [41] and vice versa [44, 45], it could be that the lack of an effect on p-Akt in 11SP is because of a much higher expression level of HIF1α than in 64SP. Alternatively and additionally, riluzole is not such a potent inhibitor of the pAkt/HIF pathway when compared to LY294002. Importantly, both treatments caused the downregulation of GLUT3 (Figure 3D) in both cell lines. Downregulation of both GLUT3 and HIF1α are beneficial for the BTSCs.

The decrease in p-Akt could also play a role in the observed decrease in proliferation of riluzole-treated BTSCs. It is known that the active form of the Akt protein can increase neural stem cell proliferation by increasing metabolism through glycogen synthase kinase (GSK-3) and promoting transcription and protein synthesis [46, 47]. We did not examine whether the declined proliferation was a direct response to the decreased p-Akt pathway.

Given the importance of glucose metabolism in the regulation of cell death, we examined the form of cell death caused by riluzole treatment. Mcl-1 is an antiapoptotic protein, which levels decrease when glucose becomes limited, whereas increased glucose metabolism protects Mcl-1 from degradation [48]. It has been reported that degradation of Mcl-1 can be a consequence of induced apoptosis because of the inhibition of glucose metabolism through the glycolysis/Akt pathway [49] or that nutrient deprivation, under a condition that triggers Mcl-1 degradation, causes cell death by autophagy [50]. We detected a 50% downregulation of Mcl-1 after 72 h, as well as lipidation of endogenous LC3B, suggesting there is an influence from reduced metabolism on cell death by autophagy in these cells under riluzole treatment.

The other important link is the connection between metabolism and epigenetic changes in cancer cells. Cancer cells metabolize glucose to lactate. The high production of lactate causes an imbalance in the NADH/NAD^+^ ratio that can modulate the level of histone acetylation and methylation [51]. Because the reduction of DNMT1 mRNA and protein expression was first detectable after 72 h, it is possible this was because of changes in metabolism. In turn, this change influences histone methylation and DNMT1 expression. DNMT1 is overexpressed in GBM, causing hypermethylation of various tumor suppressor genes and resulting in a lack of cell growth regulation and higher genomic instability, which eventually leads to a poor prognosis in gliomas [27]. Because the DNMT1 gene is differentially regulated by both histone acetylation and histone methylation in gliomas [27], we speculate that the change in expression of DNMT1 could be the consequence of metabolism changes under riluzole treatment. Downregulation of DNMT1 is also an important finding since it has been shown that its silencing enhances glioma chemosensitivity to temodal [52].

Keeping in mind the fact that upregulation of GLUT3 is directly associated with the acquisition of a stem cell state and the ability to propagate tumors *in vivo,* which is the gold standard for BTSCs function, we examined the ability of these cells treated with riluzole to form a tumor *in vivo*. Our results showed that treating BTSCs with riluzole leads to a reduction of tumor formation in *in vivo* CAM assay. Riluzole treatment significantly decreased *in vivo* tumor growth compared to a treatment with PBS as negative control.

Additionally, cancer stem cells have active mechanisms for radiation and chemotherapy resistance. Brain tumor recurrence has been reported to be a consequence of cancer stem cell resistance and survival [9, 53]. Glucose is essential for the phosphorylation of Akt and the resultant induction of HIF1α expression at the edges of viable regions after radiotherapy [23]. To examine if riluzole improves radiation therapy, a combination of riluzole and ionizing radiation was applied to BTSCs that were implanted in CAM. A greater reduction in tumor growth was observed compared to non-irradiated riluzole-treated cells. This aligns with a study where it was reported that riluzole enhances ionizing radiation–induced cytotoxicity in human melanoma cells [54]. The *in vivo* data indicates a much more significant effect than elucidated in the *in vitro* experiments with BTSCs. This may indicate that the anti-tumor effect of riluzole also depends on the interaction of the tumor cells with their microenvironments. Acknowledging that further studies are required, we speculate that radiation-induced improvement of glucose and oxygen availability in GBM [23] could be treated with riluzole. One suggestion for further examination could be the application of riluzole during or after treatment with radiotherapy. Moreover, because this drug is already approved for ALS and has fewer side effects than conventional chemotherapeutics temodal, it would also allow for a longer application period.

Taken together, our results showed that riluzole, an approved drug, reduces the expression of GLUT3, a molecular marker known to be important for the tumorigenic potential of BTSCs. Besides GLUT3, the active form of Akt (pAkt), HIF1α, Mcl-1, and DNMT1 were downregulated. Furthermore, proliferation and cell death, two hallmarks of cancer cells, were positively influenced by riluzole treatment. Finally, the formation of tumors *in vivo* was reduced under the influence of riluzole, suggesting this potent drug should be further examined in the treatment of glioblastoma stem cell therapy.

## MATERIALS AND METHODS

### Materials

Cell culture material was obtained from Gibco (Life Technologies, Waltham, USA). Unless stated otherwise, all chemicals were purchased from Sigma-Aldrich (St. Louis, MO, USA). Antibodies: anti-Nestin (Millipore, Billerica, USA; 1:50), anti-CD133 (Biorbyt, Cambridge, UK; 1:50), anti-MAP2 and anti-OLIG2 (Millipore, Billerica, USA; 1:50), anti-GFAP (Leica, Wetzlar, Germany; 1:50), anti-Ki-67 (DAKO, Hamburg, Germany; 1:50) anti-GLUT3 (Abcam, Cambridge, UK; 1:300), anti-HIF1α (R&D Systems, Minneapolis, USA; 1:200) anti-Akt and anti-Phospho-Akt (Cell Signaling, Danvers, USA; 1:1000), anti-Mcl-1(Santa Cruz Biotechnology, Dallas, USA; 1:100), anti-Caspase 9 (Santa Cruz Biotechnology, Dallas, USA; 1:200), anti-Caspase 3 (Cell Signaling, Danvers, USA; 1:200), anti-LC3B (Cell Signaling, Danvers, USA; 1:500), anti-DNMT1 (Cell Signaling, Danvers, USA; 1:500), 10 or 50 µM Riluzole (TOCRIS, Bristol, UK), 200 µM Temodal (TMZ, MSD Sharp & Dohme GmbH. Haar, Germany) dissolved in DMSO, LY294002 (Calbiochem, EMD Millipore, Billerica, USA).

### Cell culture

Cell lines were derived from primary glioblastoma tissue samples that were obtained from patients operated on in the neurosurgery department of the Georg-August University, Göttingen, Germany. This study was approved by the ethical board of the University Clinic of Göttingen. The tissue was mechanically minced and dissociated, and the resulting cell suspension was plated in a defined medium: NeuroBasal, supplemented with the B27 component, 20 ng/ml EGF (Biochrom, Merck KGaA, Berlin, Germany) 10 ng/ml bFGF (Biochrom, Berlin, Germany) and 10 mg/ml BSA (Roche, Mannheim, Germany). These experimental conditions promote the formation of spherical clusters, called BTSCs, from floating cultures of single cells. The cell lines were characterized by CD133 and Nestin staining as well as their ability to differentiate into the three different neural lineages and to form colonies in soft agar assay (Supplementary Information). The neurospheres were allowed to settle by gravity. The supernatant was removed, and the pellet was mixed gently with 1 ml of 4% PFA/PBS. The neurospheres were allowed to settle again in the tube, the fixative was removed, and they were rinsed with 1 ml 1X phosphate-buffered saline (1X PBS), which was repeated three times. The final PBS was completely removed, and 1 ml of 30% sucrose/PBS was added. The tubes with prepared neurospheres were placed into the fridge and allowed to settle overnight. The next day, sucrose was removed and an embedding medium (Surgipath, FSC 22 frozen section compound, Leica, Wetzlar, Germany) was added; this was mixed with a needle (gently swirling) so that the spheres were completely surrounded by the embedding medium, and then, this mixture was frozen in liquid nitrogen. Sections of neurosphers were sliced with cryostat, 10 µm thick.

### Cell treatment

Single cell suspensions of brain tumor stem-like cells were seeded in the appropriate density 24 h before the treatment with replicates for each performed test. The culture medium was supplemented with 10% fetal calf serum (FCS) to allow cells to adhere. At time point 0, the cells were washed with 1X PBS and replaced with a culture medium containing the treatment compounds (without FCS) to exclude the undefined effect of the FCS medium. The control group was treated with PBS; riluzole groups were treated with 10 or 50 µM riluzole; the Co-DMSO group was treated with DMSO additionally to mimic TMZ, which was diluted in DMSO; the TMZ group was treated with 200 µM TMZ; the TMZ + riluzole groups were treated with 200 µM TMZ and 10 or 50 µM riluzole, respectively. The LY294002 group was treated with 100 µM LY294002. The addition of compounds was repeated every 24 h. After 8 h, 24 h, or 72 h, subsequent analyses were done according to the protocol for the real-time PCR (qPCR), MTT assay, ICC, and WB.

### Cell viability assay (MTT)

The spheres were trypsinized, and 5–10 × 10^3^ cells were plated in four replicates in 96-well plates in a final volume of 100 µl per well with a medium containing 10% FCS; this was then incubated at 37°C. After 24 h, the cells were washed with 1X PBS, and a new medium (without FCS) with the appropriate treatments was applied and further incubated for 72 h before adding 3-(4, 5-dimethylthiazol)-2, 5-diphenyltetrazolium bromide (MTT; Sigma-Aldrich, St. Louis, MO, USA). After the addition of MTT, the cells were incubated for a further 4 h at 37°C. The absorptions were measured with a spectrophotometer absorption reader (Bio-TEK, Winooski, USA) via absorbance at 562 nm.

### Radiation for MTT and CAM assay

For the MTT assay, the cells were irradiated after a 24 h treatment using Siemens, RS225A X-Ray Research System with a radiation dose of 5 Gy.

### RNA purification, reverse transcription, and real-time PCR amplification

Total cellular RNA was isolated with the RNeasy kit (Qiagen, Hilden, Germany) and reverse-transcribed into cDNA using the SuperScript III first-strand synthesis kit (Life Technologies, Waltham, USA). cDNA (100 ng) was used for real-time PCR amplification. Real-time PCR was performed on a BioRAD CFX384 cycler using iTaq universal SYBR-Green Mastermix (Bio-rad, Hercules, USA) and gene-specific primers as follows: (NM_000190.3) HMBS forward: 5′ CGC ATC TGG AGT TCA GGA GTA 3′ , reverse: 5′ CCA GGA TGA TGG CAC TGA 3′; (NM_000194.2) HPRT1 forward: 5′ TGA CCT TGA TTT ATT TTG CAT ACC 3′, reverse: 5′ CGA GCA AGA CGT TCA GTC CT 3′; (NM_006931.2) SLC2A3 forward: 5′ AGC TCT CTG GGA TCA ATG CTG TGT 3′, reverse: 5′ ATG GTG GCA TAG ATG GGC TCT TGA 3′; (NM_006516.2) SLC2A1 forward: 5′ ATC GTG GCC ATC TTT GGC TTT GTG 3′ reverse: 5′ CTG GAA GCA CAT GCC CAC AAT GAA 3′; (NM_001130823.2) DNMT1 forward: 5′ CGA TGT GGC GTC TGT GAG 3′, reverse: 5′ TGT CCT TGC AGG CTT TAC ATT 3′; (NM_001009999.2) KDM1A forward: 5′ GCT CGG GCT CTT ATT CCT A 3′, reverse 5′ CCC AAA AAC TGG TCT GCA AT 3′. The amplification was carried out by using the following cycle protocol: 95°C for 15 s; 60°C for 30 s, repeating 40×. Data were normalized to HMBS or HPRT1 levels.

### Transfection of BTSC

BTSC were transfected with 1µg of plasmid HA-HIF1α-pcDNA3 (a gift from William Kaelin (Addgene plasmid # 18949)) or 1 µg of pcDNA3 (ThermoFischer Scientific, Waltham, USA) alone. At 24 h prior to transfection, cells were seeded in a medium with 10% FCS, and on the next day, the cells were washed with 1XPBS and transfected using Lipofectamine 3000 (Invitrogen, Carlsbad, CA, USA) in a culture medium without FCS, per the manufacturer’s protocol. Cell treatment was performed immediately after transfection, as described previously.

### Whole-cell protein extract and western blot analysis

One day after the cells were plated in a medium with FCS, the cells were washed with 1X PBS and exposed to the appropriate treatment for 8–72 h. The cells were scraped and lysed in a whole-cell extract buffer (150 mM NaCl, 40 mM NaF, 5 mM EDTA, 5 mM EGTA, 1mM Na_3_VO_4,_1% (v/v) Igepal, 1% (m/v) Sodium deoxycholate, pH: 8,3 and complete, EDTA-free protease inhibitor cocktail 25X (Roche, Mannheim, Germany)). After centrifugation, the protein concentrations were determined using the Lowry method. SDS-PAGE and immunoblotting were carried out using an apropriate primary antibody and anti-species-specific horseradish-peroxidase-linked secondary antibodies. Controls of protein loading were immunoblotted with an anti-β-actin (Abcam, Cambridge, UK; 1:5000). Immunoreactivity was visualised with chemiluminescence and quantified by the Quantity One analysis software (Bio-Rad, Munich, Germany).

### Cell proliferation assay and immunocytochemistry (ICC)

The spheres were trypsinized, and 4x10^4^ cells were seeded in two replicates on poly-D-lysine-coated coverslips in a medium with FCS to allow them to set. After 24 h, the medium was aspirated, cells were washed with 1X PBS, and a new medium without FCS plus the appropriate treatment was added. At 24 h after riluzole treatment, the cells were fixed with methanol for 20 min at −20°C. After blocking, the cells were incubated with the primary antibody overnight at 4°C. The cells were visualized using the Alexa Fluor 488 secondary antibody (Life Technologies, Waltham, USA; 1:500) and counterstained with DAPI (Sigma-Aldrich, St. Louis, MO, USA). Finally, the coverslips were dried at room temperature and mounted with the Aqua Polymounting medium (Polysciences, Inc., Eppelheim, Germany). The slides were examined with the ZEISS Axiovert 200 Fluorescence Microscope. The proportion of Ki-67 positive cells was counted using 20× microscopic amplification. Each treatment was performed in at least three independent experiments, and each experiment was analyzed based on at least five random microscopic fields.

### CAM assay

For the CAM assays, fertilized chicken eggs were incubated at 37°C and 80% atmospheric moisture. A small window (1 × 2 cm) was made in the eggshell of 5-day-old embryos, and the windows were sealed with adhesive tape. The chicken embryos were returned to the incubator until day 8 when the trypsinized neurosphere suspension of 2 × 10^6^ (64SP; *n* = 3 chicken embryos) was mixed with 100 μl growth factor reduced matrigel (BD Biosciences, Heidelberg, Germany) and inoculated by pipetting onto the chorioallantoic membrane of 8-day-old chicken embryos. On days 10–16, the tumors were treated with 250 µl of 10 µM or 50 μM riluzole or PBS. At 72 h after inoculation, chicken embryos were irradiated with 5 Gy. Matrigel grafts with surrounding CAM were cut out from each embryo on day 7 after sphere implantation, and these specimens were weighted.

### Statistical analysis

The results shown are the mean values of at least three independent experiments (±S.E.M.) unless expressly mentioned. The significance of differences was analyzed using two-sided *t*-tests for two-group comparisons. Calculations were performed using the statistics software Prism 5 (GraphPad Software, La Jolla, USA). A probability of *p* < 0.05 was considered statistically significant.

## SUPPLEMENTARY MATERIAL FIGURES



## References

[1] Warburg O (1956). On the origin of cancer cells. Science.

[2] Oudard S, Arvelo F, Miccoli L, Apiou F, Dutrillaux AM, Poisson M, Dutrillaux B, Poupon MF (1996). High glycolysis in gliomas despite low hexokinase transcription and activity correlated to chromosome 10 loss. Br J Cancer.

[3] Peters A, Schweiger U, Pellerin L, Hubold C, Oltmanns KM, Conrad M, Schultes B, Born J, Fehm HL (2004). The selfish brain: competition for energy resources. Neurosci Biobehav Rev.

[4] Boado RJ, Black KL, Pardridge WM (1994). Gene expression of GLUT3 and GLUT1 glucose transporters in human brain tumors. Brain Res Mol Brain Res.

[5] Vannucci SJ, Maher F, Simpson IA (1997). Glucose transporter proteins in brain: delivery of glucose to neurons and glia. Glia.

[6] Ayala FR, Rocha RM, Carvalho KC, Carvalho AL, da Cunha IW, Lourenco SV, Soares FA (2010). GLUT1 and GLUT3 as potential prognostic markers for Oral Squamous Cell Carcinoma. Molecules.

[7] Flavahan WA, Wu Q, Hitomi M, Rahim N, Kim Y, Sloan AE, Weil RJ, Nakano I, Sarkaria JN, Stringer BW, Day BW, Li M, Lathia JD (2013). Brain tumor initiating cells adapt to restricted nutrition through preferential glucose uptake. Nat Neurosci.

[8] Masin M, Vazquez J, Rossi S, Groeneveld S, Samson N, Schwalie PC, Deplancke B, Frawley LE, Gouttenoire J, Moradpour D, Oliver TG, Meylan E (2014). GLUT3 is induced during epithelial-mesenchymal transition and promotes tumor cell proliferation in non-small cell lung cancer. Cancer Metab.

[9] Bao S, Wu Q, McLendon RE, Hao Y, Shi Q, Hjelmeland AB, Dewhirst MW, Bigner DD, Rich JN (2006). Glioma stem cells promote radioresistance by preferential activation of the DNA damage response. Nature.

[10] Morfouace M, Lalier L, Bahut M, Bonnamain V, Naveilhan P, Guette C, Oliver L, Gueguen N, Reynier P, Vallette FM (2012). Comparison of spheroids formed by rat glioma stem cells and neural stem cells reveals differences in glucose metabolism and promising therapeutic applications. J Biol Chem.

[11] Zhou Y, Zhou Y, Shingu T, Feng L, Chen Z, Ogasawara M, Keating MJ, Kondo S, Huang P (2011). Metabolic alterations in highly tumorigenic glioblastoma cells: preference for hypoxia and high dependency on glycolysis. J Biol Chem.

[12] Xu CF, Liu Y, Shen S, Zhu YH, Wang J (2015). Targeting glucose uptake with siRNA-based nanomedicine for cancer therapy. Biomaterials.

[13] Lacomblez L, Bensimon G, Leigh PN, Guillet P, Meininger V, Amyotrophic Lateral Sclerosis/Riluzole Study Group II (1996). Dose-ranging study of riluzole in amyotrophic lateral sclerosis. Lancet.

[14] Doble A (1996). The pharmacology and mechanism of action of riluzole. Neurology.

[15] Urbani A, Belluzzi O (2000). Riluzole inhibits the persistent sodium current in mammalian CNS neurons. Eur J Neurosci.

[16] Frizzo ME, Dall’Onder LP, Dalcin KB, Souza DO (2004). Riluzole enhances glutamate uptake in rat astrocyte cultures. Cell Mol Neurobiol.

[17] Yelskaya Z, Carrillo V, Dubisz E, Gulzar H, Morgan D, Mahajan SS (2013). Synergistic inhibition of survival, proliferation, and migration of U87 cells with a combination of LY341495 and Iressa. PLoS One.

[18] Pittenger C, Coric V, Banasr M, Bloch M, Krystal JH, Sanacora G (2008). Riluzole in the treatment of mood and anxiety disorders. CNS Drugs.

[19] Chowdhury GM, Banasr M, de Graaf RA, Rothman DL, Behar KL, Sanacora G (2008). Chronic riluzole treatment increases glucose metabolism in rat prefrontal cortex and hippocampus. J Cereb Blood Flow Metab.

[20] Zhang C, Yuan XR, Li HY, Zhao ZJ, Liao YW, Wang XY, Su J, Sang SS, Liu Q (2015). Anti-cancer effect of metabotropic glutamate receptor 1 inhibition in human glioma U87 cells: involvement of PI3K/Akt/mTOR pathway. Cell Physiol Biochem.

[21] Hockly E, Tse J, Barker AL, Moolman DL, Beunard JL, Revington AP, Holt K, Sunshine S, Moffitt H, Sathasivam K, Woodman B, Wanker EE, Lowden PA (2006). Evaluation of the benzothiazole aggregation inhibitors riluzole and PGL-135 as therapeutics for Huntington’s disease. Neurobiol Dis.

[22] Zheng C, Yang K, Zhang M, Zou M, Bai E, Ma Q, Xu R (2016). Specific protein 1 depletion attenuates glucose uptake and proliferation of human glioma cells by regulating GLUT3 expression. Oncol Lett.

[23] Harada H, Itasaka S, Kizaka-Kondoh S, Shibuya K, Morinibu A, Shinomiya K, Hiraoka M (2009). The Akt/mTOR pathway assures the synthesis of HIF-1alpha protein in a glucose- and reoxygenation-dependent manner in irradiated tumors. J Biol Chem.

[24] Yu J, Li J, Zhang S, Xu X, Zheng M, Jiang G, Li F (2012). IGF-1 induces hypoxia-inducible factor 1alpha-mediated GLUT3 expression through PI3K/Akt/mTOR dependent pathways in PC12 cells. Brain Res.

[25] Kondo K, Klco J, Nakamura E, Lechpammer M, Kaelin WG (2002). Inhibition of HIF is necessary for tumor suppression by the von Hippel-Lindau protein. Cancer Cell.

[26] Suva ML, Rheinbay E, Gillespie SM, Patel AP, Wakimoto H, Rabkin SD, Riggi N, Chi AS, Cahill DP, Nahed BV, Curry WT, Martuza RL, Rivera MN (2014). Reconstructing and reprogramming the tumor-propagating potential of glioblastoma stem-like cells. Cell.

[27] Rajendran G, Shanmuganandam K, Bendre A, Muzumdar D, Goel A, Shiras A (2011). Epigenetic regulation of DNA methyltransferases: DNMT1 and DNMT3B in gliomas. J Neurooncol.

[28] Koch R, Demant M, Aung T, Diering N, Cicholas A, Chapuy B, Wenzel D, Lahmann M, Guntsch A, Kiecke C, Becker S, Hupfeld T, Venkataramani V (2014). Populational equilibrium through exosome-mediated Wnt signaling in tumor progression of diffuse large B-cell lymphoma. Blood.

[29] Wittig-Blaich SM, Kacprzyk LA, Eismann T, Bewerunge-Hudler M, Kruse P, Winkler E, Strauss WS, Hibst R, Steiner R, Schrader M, Mertens D, Sultmann H, Wittig R (2011). Matrix-dependent regulation of AKT in Hepsin-overexpressing PC3 prostate cancer cells. Neoplasia.

[30] Lokman NA, Elder AS, Ricciardelli C, Oehler MK (2012). Chick chorioallantoic membrane (CAM) assay as an *in vivo* model to study the effect of newly identified molecules on ovarian cancer invasion and metastasis. Int J Mol Sci.

[31] Taizi M, Deutsch VR, Leitner A, Ohana A, Goldstein RS (2006). A novel and rapid *in vivo* system for testing therapeutics on human leukemias. Exp Hematol.

[32] Balke M, Neumann A, Kersting C, Agelopoulos K, Gebert C, Gosheger G, Buerger H, Hagedorn M (2010). Morphologic characterization of osteosarcoma growth on the chick chorioallantoic membrane. BMC Res Notes.

[33] Ye ZC, Sontheimer H (1999). Glioma cells release excitotoxic concentrations of glutamate. Cancer Res.

[34] Takano T, Lin JH, Arcuino G, Gao Q, Yang J, Nedergaard M (2001). Glutamate release promotes growth of malignant gliomas. Nat Med.

[35] Rosenberg SA, Niglio SA, Salehomoum N, Chan JL, Jeong BS, Wen Y, Li J, Fukui J, Chen S, Shin SS, Goydos JS (2015). Targeting Glutamatergic Signaling and the PI3 Kinase Pathway to Halt Melanoma Progression. Transl Oncol.

[36] Grunnet M, Jespersen T, Angelo K, Frokjaer-Jensen C, Klaerke DA, Olesen SP, Jensen BS (2001). Pharmacological modulation of SK3 channels. Neuropharmacology.

[37] Richter JM, Schaefer M, Hill K (2014). Riluzole activates TRPC5 channels independently of PLC activity. Br J Pharmacol.

[38] Liu AY, Mathur R, Mei N, Langhammer CG, Babiarz B, Firestein BL (2011). Neuroprotective drug riluzole amplifies the heat shock factor 1 (HSF1)- and glutamate transporter 1 (GLT1)-dependent cytoprotective mechanisms for neuronal survival. J Biol Chem.

[39] Gottlob K, Majewski N, Kennedy S, Kandel E, Robey RB, Hay N (2001). Inhibition of early apoptotic events by Akt/PKB is dependent on the first committed step of glycolysis and mitochondrial hexokinase. Genes Dev.

[40] Rathmell JC, Fox CJ, Plas DR, Hammerman PS, Cinalli RM, Thompson CB (2003). Akt-directed glucose metabolism can prevent Bax conformation change and promote growth factor-independent survival. Mol Cell Biol.

[41] Zhong H, Chiles K, Feldser D, Laughner E, Hanrahan C, Georgescu MM, Simons JW, Semenza GL (2000). Modulation of hypoxia-inducible factor 1alpha expression by the epidermal growth factor/phosphatidylinositol 3-kinase/PTEN/AKT/FRAP pathway in human prostate cancer cells: implications for tumor angiogenesis and therapeutics. Cancer Res.

[42] Kilic M, Kasperczyk H, Fulda S, Debatin KM (2007). Role of hypoxia inducible factor-1 alpha in modulation of apoptosis resistance. Oncogene.

[43] Garcia-Echeverria C, Sellers WR (2008). Drug discovery approaches targeting the PI3K/Akt pathway in cancer. Oncogene.

[44] Qiang L, Wu T, Zhang HW, Lu N, Hu R, Wang YJ, Zhao L, Chen FH, Wang XT, You QD, Guo QL (2012). HIF-1alpha is critical for hypoxia-mediated maintenance of glioblastoma stem cells by activating Notch signaling pathway. Cell Death Differ.

[45] Semenza GL (2003). Targeting HIF-1 for cancer therapy. Nat Rev Cancer.

[46] Maurer MH, Bromme JO, Feldmann RE, Jarve A, Sabouri F, Burgers HF, Schelshorn DW, Kruger C, Schneider A, Kuschinsky W (2007). Glycogen synthase kinase 3beta (GSK3beta) regulates differentiation and proliferation in neural stem cells from the rat subventricular zone. J Proteome Res.

[47] Miyashita K, Kawakami K, Nakada M, Mai W, Shakoori A, Fujisawa H, Hayashi Y, Hamada J, Minamoto T (2009). Potential therapeutic effect of glycogen synthase kinase 3beta inhibition against human glioblastoma. Clin Cancer Res.

[48] Alves NL, Derks IA, Berk E, Spijker R, van Lier RA, Eldering E (2006). The Noxa/Mcl-1 axis regulates susceptibility to apoptosis under glucose limitation in dividing T cells. Immunity.

[49] Zhao Y, Wieman HL, Jacobs SR, Rathmell JC (2008). Mechanisms and methods in glucose metabolism and cell death. Methods Enzymol.

[50] Germain M, Nguyen AP, Le Grand JN, Arbour N, Vanderluit JL, Park DS, Opferman JT, Slack RS (2011). MCL-1 is a stress sensor that regulates autophagy in a developmentally regulated manner. EMBO J.

[51] van Horssen R, Willemse M, Haeger A, Attanasio F, Guneri T, Schwab A, Stock CM, Buccione R, Fransen JAM, Wieringa B (2013). Intracellular NAD(H) levels control motility and invasion of glioma cells. Cell Mol Life Sci.

[52] Shervington A, Patel R (2008). Silencing DNA methyltransferase (DNMT) enhances glioma chemosensitivity. Oligonucleotides.

[53] Li L, Neaves WB (2006). Normal stem cells and cancer stem cells: the niche matters. Cancer Res.

[54] Khan AJ, Wall B, Ahlawat S, Green C, Schiff D, Mehnert JM, Goydos JS, Chen S, Haffty BG (2011). Riluzole enhances ionizing radiation-induced cytotoxicity in human melanoma cells that ectopically express metabotropic glutamate receptor 1 *in vitro* and *in vivo*. Clin Cancer Res.

