# Supplementary material for: Riluzole: a potential therapeutic intervention in human brain tumor stem-like cells
Source: Oncotarget. 2017 May 20;8(57):96697–709. doi: 10.18632/oncotarget.18043 (PMC5722515; doi:10.18632/oncotarget.18043)
Supplement: Supplementary file 1 [file oncotarget-08-96697-s001.pdf]

# Riluzole: A potential therapeutic intervention in human brain tumor stem-like cells

## Supplementary Materials

### Differentiation of BITCs and immunocytochemistry

In order to identify differentiation potential, neurospheres were dissociated and seeded on coverslips coated with poly-D-Lysine (Sigma-Aldrich, St. Louis, MO, USA) in differentiation medium (culture medium with 10% FCS). After 10 days cells were fixed in 4% PFA, and incubated overnight with the following primary antibodies: anti-MAP2 (Millipore, Billerica, USA; 1:50), anti-GFAP (Leica, Wetzlar, Germany; 1:50), anti-OLIG2 (Millipore, Billerica, USA; 1:50). After washing, cells were incubated with secondary antibodies: biotinylated anti-mouse or anti-rabbit (Jackson ImmunoResearch Laboratories, West Grove, PA, USA; 1:500) and Streptavidin Alexa 488 (Life Technologies, Waltham, USA; 1:500) at room temperature. Nuclei were counterstained with DAPI. Image acquisition was done with 40×.

### Soft agar assay

Single-cell suspension of $4 \times 10^3$ cells was plated in medium containing 0.4% agar noble (Difco, Becton Dickinson and Company, Franklin Lakes, New Jersey, USA), and after that seeded in 24-well plates already containing 1, 2% agar. Cells were cultured for 3–4 weeks. The number of wells with tumorspheres was scored after 28 days by counting the colonies.

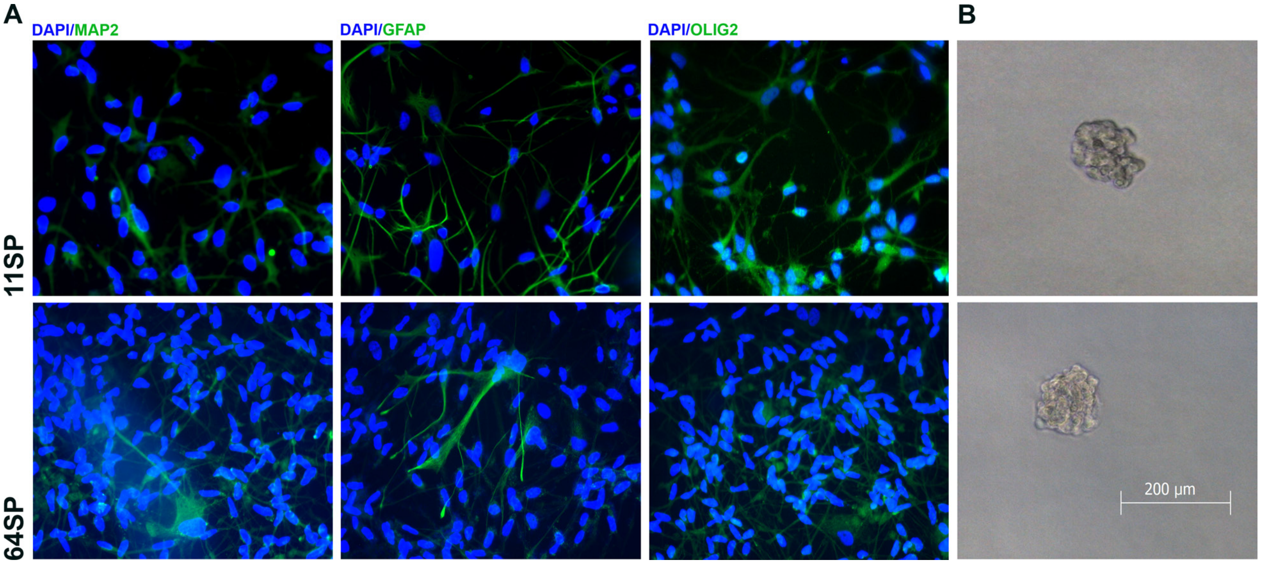


**Supplementary Figure 1: Characterization of BTSCs.** (**A**) Immunofluorescent staining of differentiated 11SP and 64SP BTSCs with neural (MAP2), astrocytes (GFAP), and oligodendrocytes (OLIG2) marker. (**B**) Representative picture of neurosphere formation from 11SP and 64SP in soft agar assay after 28 days.

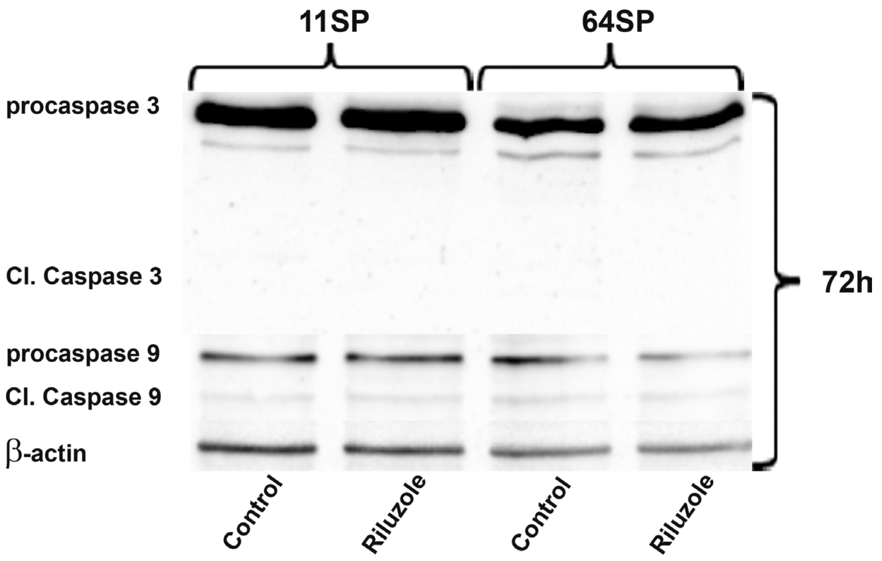


**Supplementary Figure 2: Caspase 3 and Caspase 9 expression analysis after riluzole treatment.** Whole cell lysates of 11SP and 64SP BTSCs treated with riluzole (50 μM) for indicated time was collected and analyzed by western blot. Activation of Caspase 3 and Caspase 9 was not detected. β-actin was used as loading control.
